# Automated real-time quantification of group locomotor activity in *Drosophila melanogaster*

**DOI:** 10.1038/s41598-019-40952-5

**Published:** 2019-03-14

**Authors:** Kristin M. Scaplen, Nicholas J. Mei, Hayley A. Bounds, Sophia L. Song, Reza Azanchi, Karla R. Kaun

**Affiliations:** Department of Neuroscience, Brown University Providence, Providence, USA

## Abstract

Recent advances in neurogenetics have highlighted *Drosophila melanogaste*r as an exciting model to study neural circuit dynamics and complex behavior. Automated tracking methods have facilitated the study of complex behaviors via high throughput behavioral screening. Here we describe a newly developed low-cost assay capable of real-time monitoring and quantifying *Drosophila* group activity. This platform offers reliable real-time quantification with open source software and a user-friendly interface for data acquisition and analysis. We demonstrate the utility of this platform by characterizing ethanol-induced locomotor activity in a dose-dependent manner as well as the effects of thermo and optogenetic manipulation of ellipsoid body neurons important for ethanol-induced locomotor activity. As expected, low doses of ethanol induced an initial startle and slow ramping of group activity, whereas high doses of ethanol induced sustained group activity followed by sedation. Advanced offline processing revealed discrete behavioral features characteristic of intoxication. Thermogenetic inactivation of ellipsoid body ring neurons reduced group activity whereas optogenetic activation increased activity. Together, these data establish the fly Group Activity Monitor (flyGrAM) platform as a robust means of obtaining an online read out of group activity in response to manipulations to the environment or neural activity, with an opportunity for more advanced post-processing offline.

## Introduction

Locomotor activity is a fundamental feature of nearly all behaving organisms. Analysis of activity has revealed great insight into complex behaviors as well as the underlying neural mechanisms^1–5^. However, analyses are often limited by available technologies that can be computationally expensive, time consuming, or offer poor spatial resolution. Here we introduce a new behavioral apparatus, the fly Group Activity Monitor (flyGrAM). This relatively inexpensive behavior apparatus provides an alternative to studying *Drosophila* locomotor activity by combining excellent spatial resolution, ease of use, and real-time activity analysis with open source software.

Current methods for characterizing locomotor activity fall under two broad categories: infrared (IR) beam break and video-based analyses. Beam break based methods, such as the *Drosophila* Activity Monitor (DAM)^6^, rely on occlusion of an IR beam situated across the center of a behavioral chamber to detect fly motion and activity. These methods have the advantage of being extremely scalable and are widely used, but suffer from poor spatial resolution and underestimate the amount of locomotor activity^7^. Newer video-based analysis methods use computational vision techniques to track and quantify animals’ activity. These methods are often less expensive than their beam break counterparts and provide richer behavioral readouts such as position and velocity. PySolo^8^ is an example of multiple singly housed fly video tracking that is used in *Drosophila* sleep research and popular examples of group tracking and advanced behavioral analysis include C-trax^9^ and JAABA^10^. A compendium of other video tracking solutions can be found in Table 1. Although video-based methods can analyze multiple singly housed flies in or near real-time, current methods for analysis of group housed fly activity are computationally expensive and time consuming.

**Table 1:** A sample of different video-based software methodologies for fly activity quantification.

Software	Utility
Ethovision^55^	Commercially available software; uses multi point detection to classify behaviors based off user-defined parameters. Used most frequently in rodent studies, however, recently utilized for Fly Stampede 2.0 data analysis^56^.
DIAS^26–28^	Software used to quantify movement parameters such as speed and heading offline. Note: this software is not open source and no longer actively maintained.
ActualTrack^57^	Commercial software designed to quantify detailed locomotor activity and zonal statistics of animals from video of experiments. Exports results in.xls or.csv formats.
SwisTrack^58^	Open source tracking system that uses combination of components selected and configured by user to optimize tracking of objects in real time or offline.
Motmot + flytrax^59^	Open source Python based software that allows for real-time tracking and analysis of a single walking fruit fly.
Ctrax^9^	Open source tracking that estimates position and orientation of individual flies within a group setting while maintaining individual identities.
CADABRA^60^	Open source tracking system that classifies individual component social behaviors of fly pairs based on location, orientation, and wing posture. Used primarily for measuring aggression and courtship.
Inan *et al*.^61^	Tracking system that uses one or more cameras to track active and inactive periods of populations of flies during locomotion or flight.
Buridan^62^	Open source tracking system of a single fly in the Buridan paradigm, a free choice assay between two visual landmarks, using R based centroid trajectory analysis.
IowaFLI Tracker^63^	Matlab based video analysis system used to track social interactions of multiple flies in small arena.
GroupScan^64^	Commercial software optimized for high throughput activity level screening of groups of a wide range of animals. Detectable behaviors include total average speed, live counts and visible counts.
pySolo^8^	Software used to analyze behavior of multiple singly housed flies for the analysis of sleep and locomotor activity.
Woods *et al*.^65^	Tracking system that monitors spontaneous locomotor activity of flies across several days.
Cheng *et al*.^66^	Matlab based analysis system that uses multiple synchronized cameras to track swarms of flies in 3D space.
The tracker program^7^	Open source Java based program that uses background subtraction to measure activity levels of flies. Developed to resolve overestimations of sleep in the DAM system.
DART^67^	Open source program, which reports positional and locomotor activity data of individual flies in multiple chambers (authors report that program can also track multiple animals per chamber).
FlyPi^68^	Open source system based on a Raspberry Pi computer, camera, and Arduino that emphasize low cost and flexibility. Position, orientation and other behaviors are identified and analyzed offline.
flyGrAM*	**Open source Python based system that uses background subtraction to monitor real-time group locomotor activity in multiple chambers, and emphasizes low cost, flexibility, and ease of use**.

*All software and data can be downloaded from the flyGrAM repositories (https://github.com/kaunlab).

Here, we introduce, flyGrAM, an open-source software platform and inexpensive video-based behavioral apparatus that allows for real-time quantification of group activity in *Drosophila*. Unlike advanced, but computationally expensive, video-based group analyses, the flyGrAM does not track location, velocity, or identity of any individual flies in a group. In return, it is able to combine the excellent spatial resolution of video-based methods with ease of use and an instant activity readout. Importantly, it does so with a convenient and accessible user interface for data acquisition and analysis. Further, because flyGrAM produces both analyzed activity and raw video recordings, this method provides a foundation for offline advanced post processing using C-trax^9^ and JAABA software^10^. Using a simple custom-built arena for the flyGrAM, we can easily and reliably quantify how activity of a group changes following manipulations to the environment or neural activity. Here, we quantify group activity in response to increasing doses of vaporized ethanol, characterize discrete behavioral responses to ethanol exposure, and compare differences in group activity across wildtype animals. We also quantify group activity in response to thermogenetic inactivation and optogenetic activation of ellipsoid body neurons important for ethanol induced locomotor activity^11^.

## Materials and Methods

### Fly Stocks and rearing conditions

The following fly strains were used: wild-type *Canton-S (w* + CS), *Berlin (w* + B), *OregonR (w* + O) *w- Canton-S (w* − CS*), 4-67-GAL4* from the P[GAL4GawB] collection of Dr. U. Heberlein (Janelia Research Campus), *pJFRC100-20XUAS-TTS-shibire*^*ts1*^*-p10 (VK00005; shi*^*ts*^*)*^12^, ‘empty-Gal4’ *pBDPGAL4.1Uw (attP2; pBDP-GAL4)*^13^ and *20XUAS-IVS-CsChrimson-mVenus (attP18, Chrimson)*^14^. Wild-type strain *w* + CS was used for dose response characterization. To express *Chrimson* in ellipsoid body neurons, we crossed *UAS-Chrimson* virgin female flies to flies containing *4-67-GAL4*. UAS-control flies were obtained by crossing *Chrimson* virgin female flies to *pBDP-GAL4* male flies*. GAL4* control flies were obtained by crossing *4-67-GAL4* to *w* *−* *CS* flies. To express *shibire*^*ts1*^ in ellipsoid body neurons, we crossed *UAS-shi*^*ts1*^ virgin female flies to flies containing *4-67*-*GAL4*. UAS-control flies were obtained by crossing *UAS-shi*^*ts1*^ to *yw* flies whereas *GAL4* control flies were obtained by crossing *4-67-GAL4* to *yw* flies. Flies were raised on standard cornmeal agar food media with tegosept anti-fungal agent. *w* + CS wildtype flies used for dose response experiments were maintained at 24 °C and 65% humidity and on a 14/10 hr light/dark cycle. All other flies were transitioned to 18 °C and 65% humidity following collection (2–4 days prior to testing). All male flies were collected under light, humidified, CO_2_ anesthesia 1–2 days after eclosion and 2–4 days prior to testing. For optogenetic experiments, flies were raised on 0.2 mM all-trans-retinal (ATR, Sigma-Aldrich, St. Louis, MO, USA) and transitioned to 0.4 mM ATR media post-eclosion 3–4 days prior to testing. To protect retinal from light and limit nonspecific activation of *Chrimson* expressing neurons, vials were wrapped in aluminum foil and maintained in dark conditions.

### Fabrication of behavioral chamber and flyGrAM apparatus

**Figure 1:**
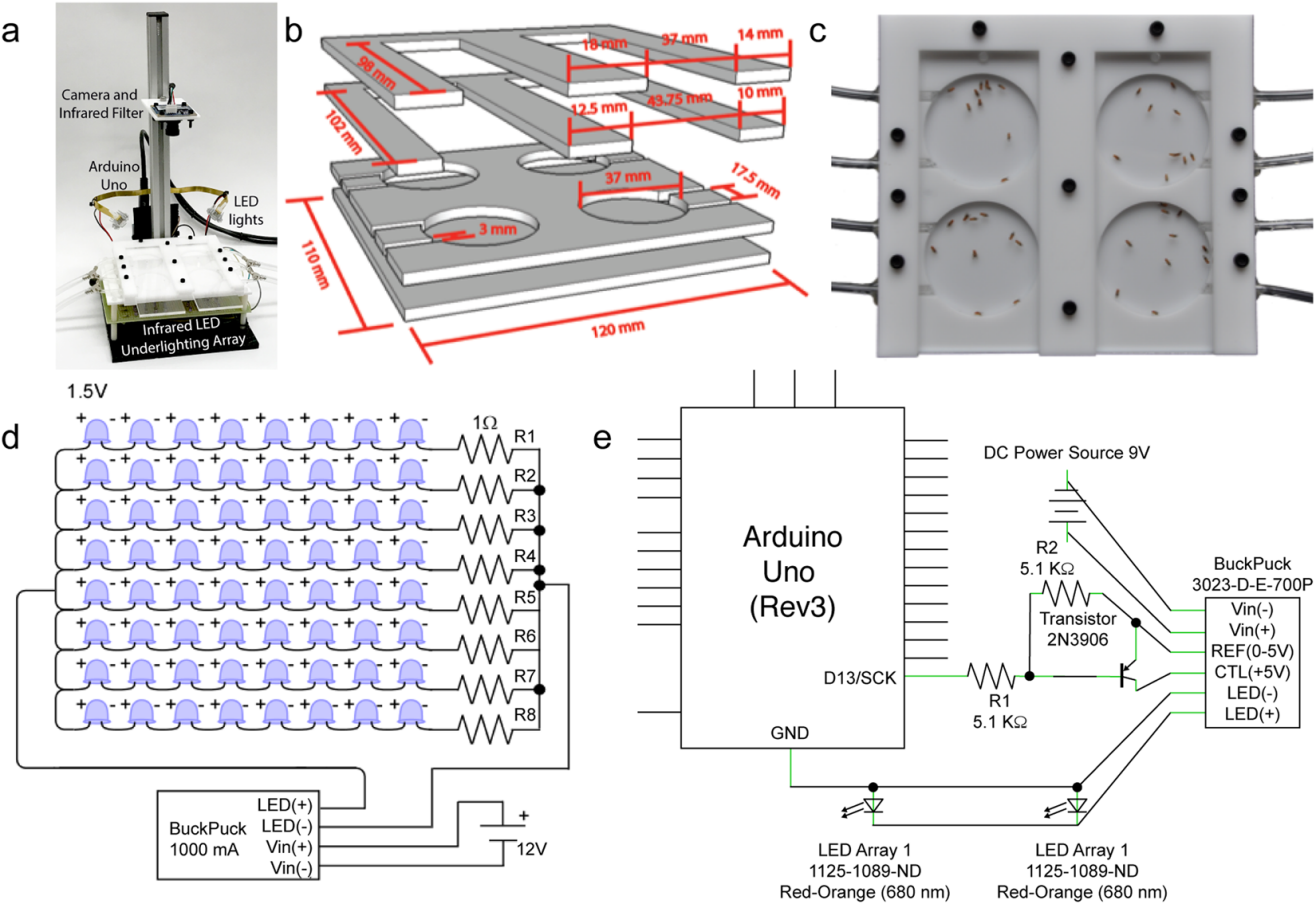
flyGrAM apparatus construction. (**a**) The fully assembled flyGrAM apparatus consists of a behavioral chamber, underlighting array, two LED arrays for optogenetic stimulation experiments (680 nm), an Arduino Uno, a USB camera, and a scaffold for securement. (**b**) A blown-out schematic of the behavioral chamber consisting of four laser cut acrylic layers. (**c**) A fully assembled behavioral chamber with loaded flies contained by 2 separate clear acrylic covers. Each individual arena was connected to an air and vacuum source via separate tubes to facilitate airflow. (**d**) Wiring schematic for infrared LED illumination array. (**e**) Detailed wiring diagram for Arduino control of optogenetics LED stimulation.

A list of minimum components and cost for constructing the flyGrAM apparatus (Fig. 1a) can be found in Table 2 (see Supplementary Table 1 for a detailed list of components). For all experiments, a custom-built four-arena (4 × 37 mm diameter circles) behavioral chamber was used, which allowed for the introduction of odors and/or vaporized ethanol (Fig. 1b,c). To fabricate the chamber, opaque white acrylic (Plastics 2000 Acrylic-TL-White-7328, Plastics 2000, Modesto, California) and clear acrylic sheets (Acrylic-TP-Clear-0000, Plastics 2000, Modesto, California) were laser cut according to a schematic PDF file (Supplementary Fig. 1). Laser cut components were assembled and fastened with nylon screws and hex nuts (McMaster-Carr, Princeton, NJ) and separated into four compartments (Fig. 1b). Finally, 3 mm diameter PTFE plastic tubing covered with a fine mesh was installed into openings leading to each arena and held in place with hot melt adhesive (3033002 Hot Glue Sticks, Gorilla, USA). All chambers were connected to an air source and passive vacuum to facilitate flow through each chamber. Assembled behavioral chambers had approximately 3 mm of headspace to prevent flight but allows for free walking (Fig. 1c).

**Table 2:** Essential components for flyGrAM software.

Component	USD 2018
Behavior Arena	$40
Long-pass Camera IR Filter	$10
IR LED Array and Heat Sink	$70
Optogenetic LED Array	$25
USB Camera (30fps, Open CV compatible)	$50
Adapter Lens	$10
Dual core CPU Computer (2 GHz 8 GB)	$500

The fully assembled flyGrAM apparatus, illustrated in Fig. 1a, consists of an under-lighting array, two overhead 680 nm septuplet LED arrays (Marktech Optoelectronics, Latham, New York) for optogenetic stimulation experiments, an Arduino Uno (Arduino Foundation), a USB camera (USBFHD01M-L21, ELPCCTV, China), and a scaffold (MB Kit System Inc., Akron, Ohio) to secure all the aforementioned components.

An infrared (IR) LED under-lighting array was constructed from 880 nm IR LEDs (SFH 487-2, Osram, Munich, Germany) arranged in 8 × 8 patterns (64 total LEDs) and wired to a 1000 mA BuckPuck (3023-D-E-1000P, LuxDrive, Randolph, Vermont) with a potentiometer connected to the REF and CTL terminals. Detailed wiring diagrams for the light arrays can be found in Fig. 1d. The potentiometer was dialed down such that just enough current was allowed to pass through for the LED array to light up, preventing LED accumulation of heat over time. A white opaque acrylic light diffuser (Plastics 2000 Acrylic-TL-White-7328-0.118″−12 × 12, Plastics 2000, Modesto, California) was mounted 2 cm above the infrared LED array and the behavioral chamber was placed on the diffuser.

Video recordings were taken with a USB camera (USBFHD01M-L21, ELP, China) positioned 10 cm above the behavioral chamber. The camera was fitted with a 6 mm focal length, F2.0, M12 adaptor lens for 1/3″ image sensors (PT-0620, M12 Lenses, Maitland, Florida). Finally, the lens was fitted with a low-wavelength pass (IR) light filter (54–664 (RG-850), Edmund Optics, Barrington, New Jersey) to prevent ambient light and optogenetic LED stimulation from adversely affecting IR tracking accuracy. The resolution of this camera is suitable for tracking flies and larvae at all stages; however, slight adjustments should be made to tracking software to decrease double detections during larval peristaltic crawling.

For optogenetic stimulation experiments a 1000 mA BuckPuck (3023-D-E-1000, LuxDrive, Randolph, Vermont) was connected to an Arduino Uno, two 5.1 K Ohm resistors, a 2N3906 transistor, as well as two 680 nm septuplet LED arrays. The stimulation LED arrays were secured to a heat-sink with plastic screws (McMaster-Carr, Princeton, NJ) and mounted above the behavioral chambers out of the camera field of view. Detailed wiring diagrams for the optogenetic stimulation LED arrays can be found in Fig. 1e.

### Detecting group locomotor activity with the flyGrAM software

**Figure 2:**
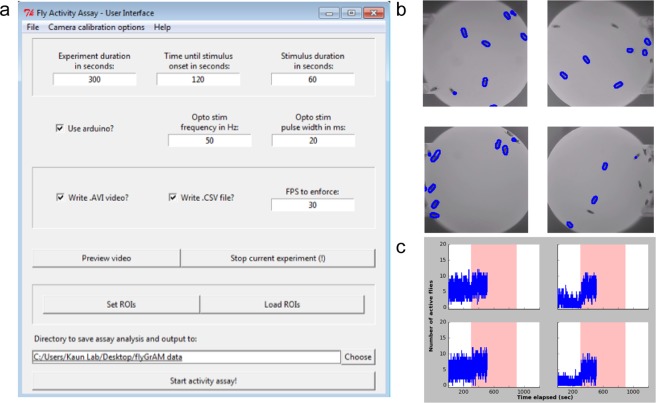
flyGrAM software: (**a**) flyGrAM User Interface where user can specify their experimental parameters (**b**) Video frame from an activity experiment, which highlights detected activity with a blue overlay around moving flies from each ROI. (**c**) Real-time analysis portion of flyGrAM software that tracks active flies and generates real-time activity plots for each ROI.

FlyGrAM was run on a desktop computer with Windows OS and a minimum of 8 GB of RAM and a dual core 2.1 GHz processor. Prior to group activity quantification, a one-time image correction procedure in the OpenCV library^15^ and Bouget (Matlab Calibration Tool)^16^ was used to remove lens barrel distortion effects. This feature is implemented in the flyGrAM’s user-friendly interface (Fig. 2a). Four regions of interest (ROI) were then manually selected and acquisition of images began (note: the number of ROIs can be increased). Videos were acquired and recorded at [640] × [480] resolution and at ~30 frames-per-second using the flyGrAM software.

Activity was detected and quantified using the fly Group Activity Monitor (flyGrAM) software, an open source, custom written Python program utilizing the free OpenCV machine vision library (http://opencv.org available for download at https://github.com/kaunlab). The flyGrAM software is written so that raw data collection occurs in parallel with group activity quantification and plotting. This ensures stable data collection performance even in computationally demanding situations where the real-time analysis cannot keep up.

For each frame acquired, a K-nearest neighbors background/foreground segmentation function^17^ from the OpenCV library was used to threshold moving flies. Subsequently, a Gaussian blur and morphological closing and opening operations were performed on the extracted foreground pixels to consolidate detected features and reducing false positives and negatives. Finally, a contour detection algorithm^18^ from the OpenCV library was used to find and count the number of consolidated foreground pixels as a measure of active flies. The output of this analysis is displayed in real-time with detections highlighted by a blue overlay around each moving fly within each ROI screen capture. Real-time activity plots for each ROI are also generated as number of active flies across time (Fig. 2c).

The flyGrAM software produces three outputs. The first output is a display of real-time detection highlighted by a blue overlap around each moving fly within each ROI screen capture and a real-time updated activity plot showing number of moving flies per ROI (Fig. 2c). This plot provides a raw visual readout of group locomotor activity during an experiment. The second output of the software is a comma separated value (CSV) file for each ROI, which can be easily exported to Excel or other popular data visualization and analysis software packages. Each CSV contains a column of experiment timestamps, number of active flies, and an optional column denoting whether an optogenetic or odor stimulus was presented during a given experimental time point. Note that due to real-time acquisition, data can be of differing lengths even when using the same time duration. This is resolved in a custom written Python based program available for download (https://github.com/kaunlab). The third and final output the software produces are raw. avi files of the entire experiments, which are amenable for Ctrax^9^ and can thus be passed to more advanced behavioral analysis programs. The data depicted in this manuscript was acquired with 10 flies per arena; however, we have successfully tracked between 1 and 30 flies per arena, and tracked flies for at least 24 hours (Supplementary Fig. 2).

The flyGrAM software successfully counts the number of moving flies in a given frame with high fidelity. In rare cases, however, two moving flies that are too close can be merged into a single detection, or single flies which make sudden decelerations can be separated into two detections (Supplementary Video 1). To address the accuracy of the flyGrAM software, we sampled randomly selected 5 seconds (150 frames) clips from 15 different arena videos used in ethanol or optogenetic experiments and compared flyGrAM group activity counts with counts derived from a human annotator. Ground truth measurements indicated that the flyGrAM software was able to achieve an average accuracy of 98.2% with a mean error rate of 1.74 ± 0.41%.

Currently, there is also no size inclusion or exclusion criterion that precludes the application of flyGrAM software from detection and analysis of locomotor activity in other organisms, including larvae. In fact, we have successfully detected movement and tracked all larval stages of *Drosophila*. However, due to stereotyped larval movement patterns duplicate detections are more frequent. Thus, we recommend the addition of a minimum size inclusion criterion for larval group activity measurements.

### Characterizing ethanol induced locomotor activity

Ethanol behavior experiments were conducted in a light sealed box to minimize the influence of external visual cues. The behavior chamber was connected to an ethanol/air delivery system through a series of three 150 mm correlated flow meters (Cole Parmer, Vernon Hills, IL)^5^. Either humidified air or differing concentrations of vaporized ethanol was introduced to all four behavioral chambers simultaneously. To achieve differing concentrations of ethanol vapor, air was first bubbled through 95% ethanol at controlled rates (40, 50, 60, 70, 80, 90, 100, or 110) and mixed with humidified air (75, 65, 55, 45, 35, 25, 15, or 5) before delivery. Overall flow rate into the behavioral chamber was kept at ~1865 ml/min (~466 ml/min per arena). In each arena, 10 male flies were added and allowed to acclimate for 15 minutes (n = 1 comprises 10 flies). Each ethanol experiment lasted 20 minutes with 5 minutes of initial baseline, 10 minutes of ethanol exposure, and a final 5 minutes of recovery. After each experiment, flies were removed and the behavioral chamber was cleaned with damp Kimwipe. Group activity changes in response to ethanol administration were averaged over 10 second bins and plotted against time.

For thermogenetic and wildtype characterization experiments ethanol concentrations were maintained at 60:55 ethanol:air ratios. Wildtype characterization experiments were performed as described above. Thermogenetic experiments were conducted in a light sealed box heated to 30–31 °C. Male flies of each genotype were added to the arena (n = 1 comprises 10 flies) and allowed to acclimate in a 30 °C incubator for 10 minutes and then the flyGrAM chamber for an additional 15 minutes. Each ethanol experiment lasted twenty minutes with 5 minutes of initial baseline, 10 minutes of ethanol exposure, and a final 5 minutes of recovery. Group activity changes in response to ethanol administration were averaged over 10 second bins and plotted against time.

### Characterizing optogenetically driven locomotor activity

Optogenetic behavior experiments were run in a light sealed box to avoid unintended photoactivation of *Chrimson* expressing neurons. Flies were raised on standard cornmeal-based medium with 0.2 nM all-*trans*-retinal. After eclosion male flies were collected and incubated for at least three days on standard cornmeal-based medium with 0.4 nM all-*trans*-retinal prior to testing. In each arena, 10 male flies were introduced to the behavioral chambers under low light conditions and given 15 minutes to habituate prior to testing (n = 1 comprises 10 flies per arena). Using the flyGrAM software, optogenetic stimulation light intensity and frequency were set by specifying LED pulse-width and frequency. Modulating the pulse-width serves as an alternative way to control the intensity of light emitted from LEDs without conventional DC voltage regulation^19^. Each experiment lasted nine minutes: 2 initial minutes of baseline activity, 5 minutes of either optogenetic stimulation with 680 nm of light or optogenetic stimulation plus ethanol exposure, and 2 final minutes of recovery. Group activity was averaged over 1 second bins, normalized to startle (30 seconds following the onset of light) by subtraction, averaged over 10 second bins and plotted against time (Fig. 7).

### Statistical Analysis

Repeated Measures ANOVAs were performed on all data sets with planned contrasts. Mauchly’s test of sphericity was first used to test for homogeneity of variance across doses or genotypes. If Mauchly’s tests were significant, indicating that the assumption of sphericity had been violated, multivariate tests were reported and degrees of freedom were corrected using Greenhouse-Geisser estimates of sphericity. Significant Repeated Measures ANOVAs were followed by posthoc tests with Bonferroni corrections. All statistical analyses were performed using SPSS software and graphs were generated with R Studio using ggplot2 plotting system.

For wildtype response to light characterization experiments startle was measured as 30 seconds following the onset of light (120–150 s). Group activity was averaged across those 30 seconds and compared to 30 seconds of baseline group activity (40–70 s). Similarly offset was defined as 30 seconds following the offset of light (420–450 s) and compared to 30 seconds of subsequent baseline activity (500–530 s). Two-tailed, one sample t-tests were performed on group activity differences from baseline at onset and offset.

## Results

Using the flyGrAM platform, we first sought to 1) understand how different vaporized ethanol concentration affects group activity across time and 2) identify how responses to ethanol differ in wild type strains. Wolf *et al*.^5^ report that at the peak of ethanol-induced hyperactivity, ethanol concentrations in whole fly extracts is approximately 20 mM. In our flyGrAM similar ethanol concentrations were measured with an ethanol:air ratio of 60:55. Thus a 60:55 ethanol: air concentration was used for characterizing wildtype differences and thermogenetic experiments. In order to quantify how increasing ethanol concentrations influenced group activity, we stepped through a variety of different ethanol: air concentrations above and below 60:55.

### Ethanol induced changes in locomotion

Consistent with other previous studies, group activity was significantly affected by the treatment of ethanol. (Repeated Measures ANOVA with planned contrasts at 140 s, 300 s, 350 s, 600 s, 800 s, and 1100 s, F(3.896, 342.853) = 182.770, p = 0.00)^5,20^. Mauchly’s test indicated that the assumption of sphericity had been violated (χ^2^(14) = 67.226, p = 0.00), therefore multivariate tests are reported (ε = 0.779).

**Figure 3:**
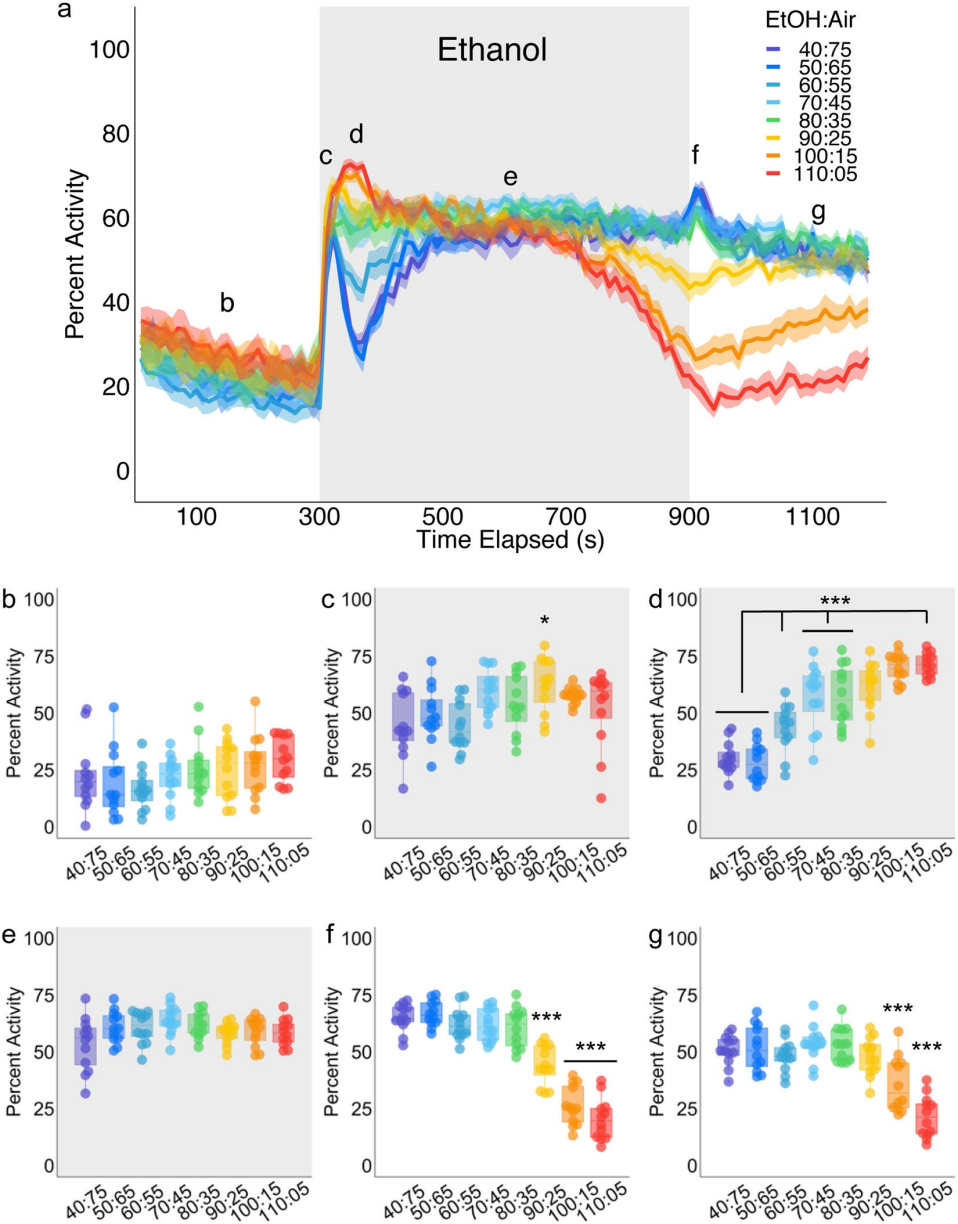
Ethanol dose effects on group locomotor activity. (**a**) Group activity counts for each dose were binned over 10 second periods, averaged across biological replicates of 10 flies each (n = 12), and plotted against time for different ethanol doses. Ethanol was delivered over a 10-minute period starting at 300 s as denoted by the gray shaded region. Lines depict mean +/− standard error. Repeated Measures ANOVA with planned contrasts at 140 s, 300 s, 350 s, 600 s, 800 s, and 1100 s (**b**–**g**) indicate a significant interaction between group activity and ethanol dose (F(27.27, 342.85) = 21.02, p = 0.000). Mauchly’s test indicated that the assumption of sphericity had been violated (χ^2^(14) = 67.226, p = 0.000), therefore multivariate tests are reported (ε = 0.779). All posthoc analyses were performed with Bonferroni corrections. (**b**) Group activity responses during baseline was not significantly different (p = 1.000) (**c**), Group activity at ethanol onset was not significantly different across doses, except for 80:35, which was significantly different from 40:75 (p = 0.020) and 60:55 (p = 0.020) (**d**) Group activity at 350 s showed significant differences between ethanol doses: 40:75 and 50:65 were not significantly different from each other (p = 1.000) but were significantly different from the remaining doses (p = 0.010–0.000). 60:55 was significantly different from all ethanol doses (p = 0.002–0.035), except for 40:75 (p = 0.051). 70:45, 80:35 were not significantly different from each other (p = 1.000) but were different from all of the lower doses and the highest dose (110:05, p = 0.026–0.000) 90:25 and 100:15 were not significantly different from each other or 70:45, 80:35, and 110:05 (p = 0.421–1.000) but were significantly different from the lower doses (40:75, 50:65, 60:55, p = 0.001–0.000). Finally, 110:05 was not significantly different from 90:25 and 110:15 (p = 0.421–1.000) but was significantly different from the lower doses (40:75, 50:65, 60:55, 70:45 and 80:35, p = 0.026–0.000). (**e**) Group activity at 600 s were not significantly different across doses (p = 0.11–1.00), except for 70:45 and 40:75, which were significantly different from each other (p = 0.015). (**f**) Group activity at 900 s for 40:75, 50:65, 60:55, 70:45, and 80:35 were not significantly different from each other (p = 1.000), however 90:25 was significantly different from all other doses (p = 0.000) and 100:15 and 110:05 were not significantly different from each other (p = 1.000) but were significantly different from the remaining doses (p = 0.000). (**g**) Group activity at 1100 s was not significantly different for nearly all doses (40:75, 50:65, 60:55, 70:45, 80:35, and 90:25, p = 1.000), however, 100:15 and 110:05 were significantly different from each other and the remaining doses (p = 0.027–0.000). *p < 0.05, ***p < 0.001.

Baseline activity levels were not significantly different across groups (Posthoc with Bonferroni correction, p = 1.000, Fig. 3b); however, ethanol elicited a consistent and significant dose-dependent set of locomotor responses. Low doses (40:75, 50:65) resulted in an initial startle and after a brief return to near baseline levels of levels, these groups increased activity and were significantly different from higher ethanol doses (60:55, 70:45, 80:35, 90:25, 100:15, 110:05; Posthoc with Bonferroni correction (p = 0.010–0.000, Fig. 3d). Interestingly, 60:55 ethanol: air dose was significantly different from the 50:65, and high ethanol doses (70:45, 80:25, 90:25, 100:15, 110:05, Posthoc with Bonferroni correction p = 0.002–0.035) but not 40:75 (Posthoc with Bonferroni correction p = 0.051). High ethanol doses resulted in a significantly higher post-startle response, sustained group activity and decreased activity after ~700 s. 90:25 and 100:15 were not significantly different from each other or 70:45, 80:35, and 110:05 (p = 0.421–1.000) but were significantly different from the lower doses (40:75, 50:65, 60:55, p = 0.001–0.000). The highest ethanol dose (110:05) was not significantly different from 90:25 and 100:15 (Posthoc with Bonferroni correction p = 0.421–1.000) but was significantly different from the lower doses (40:75, 50:65, 60:55, 70:45 and 80:35, Posthoc with Bonferroni correction p = 0.026-0.000).

Post ethanol exposure activity was also significantly different across doses. When ethanol delivery was turned off and humidified air was reintroduced, low and moderate doses (40:75, 50:65, 60:55, 70:45, 80:35) experienced a brief spike in activity, but maintained their activity levels, whereas group activity in the highest ethanol doses (90:25, 100:15, and 110:05) continued to decrease significantly (Fig. 3f). 90:25 was significantly different from all other doses (p = 0.000) and 100:15 and 110:05 were not significantly different from each other (p = 1.000) but were significantly different from the remaining doses (p = 0.000). By 1100 s, flies exposed to 90:25 ethanol had recovered, but group activity recorded from flies exposed to 100:15 and 110:05, remained significantly different (p = 0.027–0.000).

#### Ethanol Induced changes in behavioral features

The flyGrAM platform is a robust means of obtaining an online read out of group activity in response to experimental manipulations, but it also provides an opportunity for more advanced behavioral characterization. To further characterize more discrete behavioral responses to ethanol we used the raw video recordings from flyGrAM to obtain trajectory outputs from Ctrax, a machine vision program^9^. We focused our analysis on the ethanol:air ratio of 60:55 and 30 seconds surrounding baseline (135–165 s; Fig. 3b), early ethanol exposure (305–335 s; Fig. 3d), and prolonged ethanol exposure (585–615 s; Fig. 3e).

**Figure 4:**
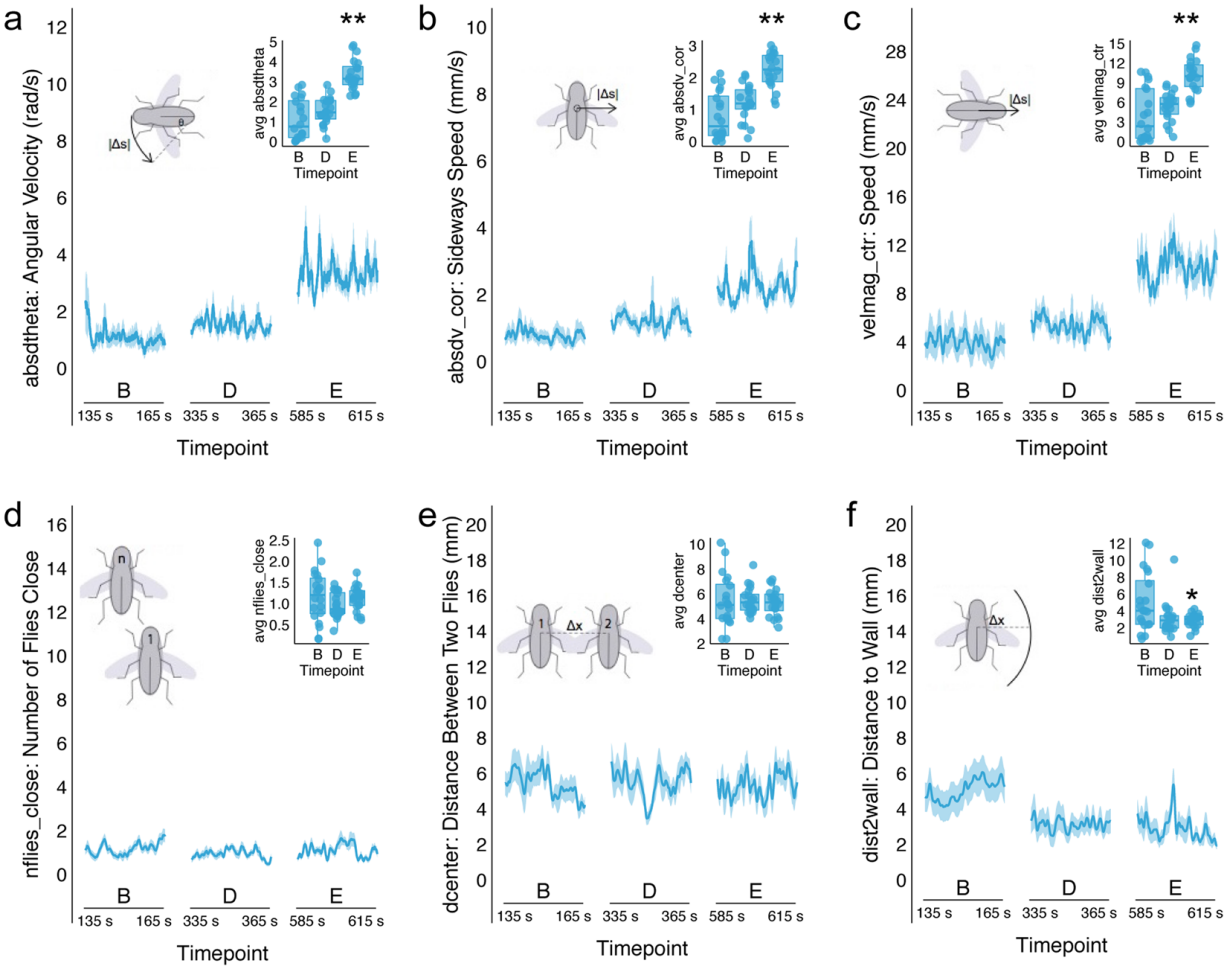
Ethanol affects discrete behavioral features in individual flies. Activity of flies were tracked and analyzed using Ctrax software from two videos during thirty seconds surrounding three timepoints selected from the 60:55 ethanol dose in Fig. 3 (b, d, and e, n = 20). Data was averaged across 1 second bins and plotted against time. Lines depict mean +/− standard error. Repeated Measures ANOVA were performed on featural data that was averaged across flies for each Timepoint (boxplot inset). (**a**) Angular velocity significantly increased following 5 minutes of ethanol exposure (Timepoint E) as compared to Timepoints B and D (Repeated Measures ANOVA F(2, 38) = 49.872, p = 0.000; Posthoc using Bonferroni correction p = 0.000). (**b**) Sideways speed also significantly increased at Timepoint E as compared to Timepoints B and D (Repeated Measures ANOVA F(2, 38) = 29.219, p = 0.000); Posthoc using Bonferroni correction p = 0.000). (**c**) Speed significantly increased at Timepoint E as compared to Timepoints B and D (Repeated Measures ANOVA F(2, 38) = 22.188, p = 0.000; Posthoc using Bonferroni correction p = 0.000). (**d**) The numbers of flies close and (**e**) distance between flies didn’t significantly change, but did significantly decrease in variability (Mauchly’s Test of Sphericity χ^2^(2) = 13.534, p = 0.001, Mauchly’s Test of Sphericity χ^2^(2) = 9.244, p = 0.01, respectively). (**f**) Proximity to the wall of the enclosure also significantly decreased variability and distance with ethanol exposure (Mauchly’s Test of Sphericity χ^2^(2) = 8.649, p = 0.013; Repeated Measures ANOVA with Greenhouse-Geisser correction F(1.448, 27.506) = 5.500, p = 0.017) Posthoc with Bonferroni correction revealed that Timepoint E was significantly different from baseline (Timepoint B p = 0.046). *p < 0.05, **p < 0.01.

Following prolonged exposure to ethanol (Timepoint E) flies significantly increased their angular velocity (Repeated Measures ANOVA F(2, 38) = 49.872, p = 0.000; Posthoc using Bonferroni correction, p = 0.000 Fig. 4a), sideways speed (Repeated Measures ANOVA F(2, 38) = 29.219, p = 0.000; Posthoc using Bonferroni correction p = 0.000 Fig. 4b) and forward speed (Repeated Measures ANOVA F(2, 38) = 22.188, p = 0.000; Posthoc using Bonferroni correction p = 0.000 Fig. 4c) suggesting that these behavioral features correlate with intoxication.

Interestingly, social features such as the numbers of flies close (Fig. 4d) and distance between flies (Fig. 4e) didn’t significantly change, however, variability in these features significantly decreased with ethanol exposure (Mauchly’s Test of Sphericity χ^2^(2) = 13.534, p = 0.001, Mauchly’s Test of Sphericity χ^2^(2) = 9.244, p = 0.01, respectively). Distance and variability in proximity to the wall of the enclosure (Fig. 4f) also significantly decreased with ethanol exposure (Mauchly’s Test of Sphericity χ^2^(2) = 8.649, p = 0.013; Repeated Measures ANOVA with Greenhouse-Geisser correction F(1.448, 27.506) = 5.500, p = 0.017). Specifically, group activity during prolonged ethanol exposure (Timepoint E) was significantly different from baseline (Timepoint B; Posthoc using Bonferroni corrections p = 0.046) suggesting that as flies become intoxicated, they remain closer to the walls of a circular enclosure.

#### Group activity responses differs across wildtype strains

**Figure 5:**
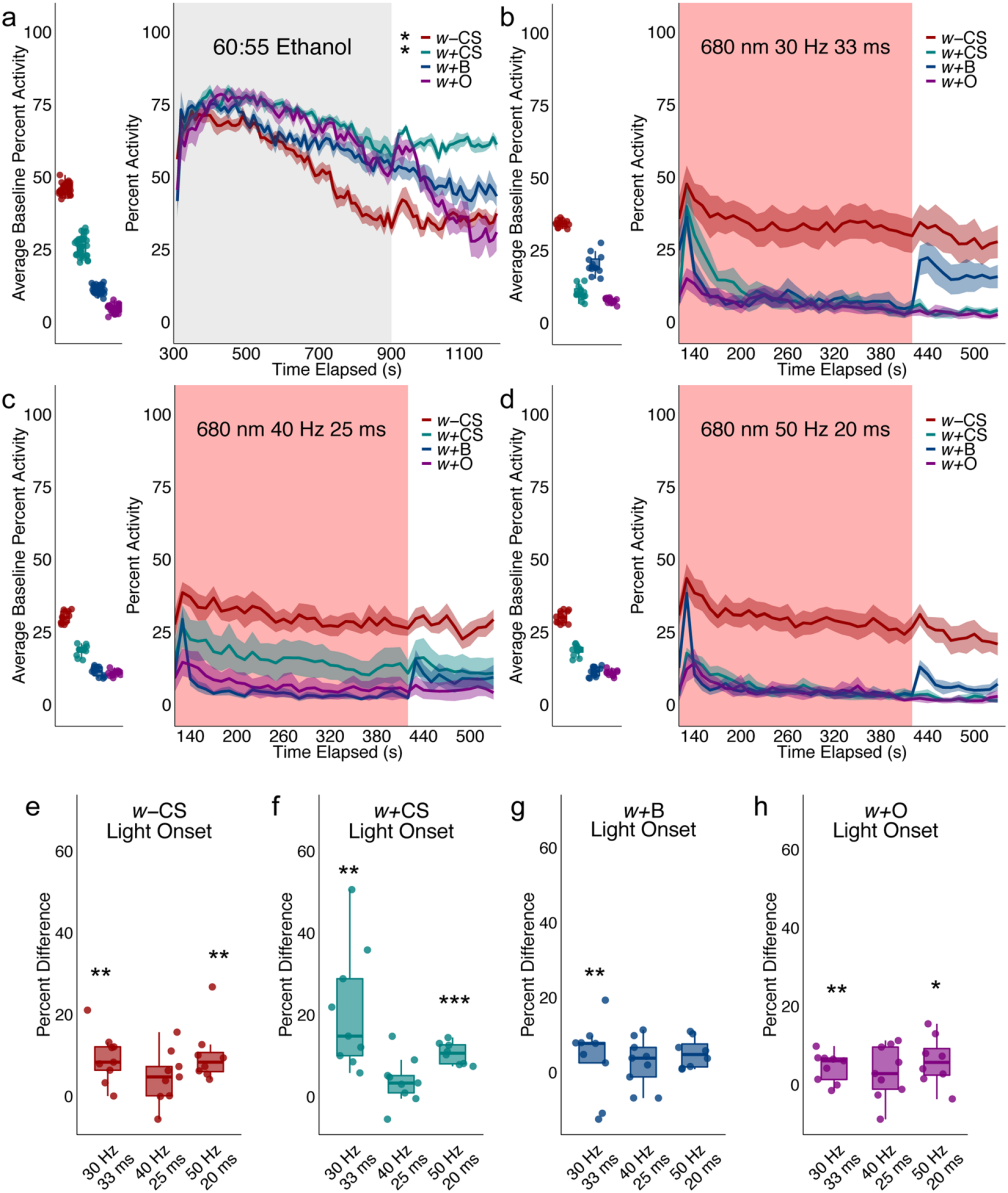
Characterization of wildtype responses to ethanol and red-light stimulation. (**a**) Group activity counts for each genotype were binned over 10 second periods, averaged across biological replicates (*w* *−* CS n = 8, *w* + CS n = 8, *w* + B n = 9, *w* + O n = 7) of 10 flies each and plotted against time. Ethanol was delivered over a 10-minute period starting at 300 s as denoted by the gray shaded region. Lines with shaded ribbon depict mean +/− standard error. Group activity during baseline was averaged across flies and plotted by genotype. Repeated Measures ANOVA with planned contrasts at 140 s, 600 s, and 1100 s revealed a significant interaction between group activity and genotype (F(6, 56) = 14.499, p = 0.00; (**a**) *w* + B and *w* + O were not significantly different from each other, however, *w* *−* CS and *w* + CS responses were significantly different from each other and the remaining wildtype groups (Posthoc with Bonferroni Corrections p = 0.033–0.000). (**b**) Wildtype responses to 680 nm of red light at 30 Hz and 33 ms (*w* *−* CS n = 9, *w* + CS n = 9, *w* + B n = 9, *w* + O n = 9). (**c**) Wildtype responses to 680 nm of red light at 40 Hz and 25 ms (*w* *−* CS n = 9, *w* + CS n = 9, *w* + B n = 9, *w* + O n = 9). (**d**) Wildtype response to 680 nm of red light 50 Hz and 20 ms (*w* *−* CS n = 8, *w* + CS n = 8, *w* + B n = 8, *w* + O n = 8). (**e**–**h**) Percent different in group activity from light onset (120–150 s) as compared to baseline (40–70 s). (**e**) *w* − CS group activity in response to 30 Hz and 33 ms and 50 Hz and 20 ms of light was significantly different from baseline (One-Sample 2-tailed T-Test t(8) = 4.576 p = 0.002, t(8) = 4.548 p = 0.002, respectively). (**f**) *w* + CS group activity in response to 30 Hz and 33 ms and 50 Hz and 20 ms of light was significantly different from baseline (One-Sample 2-tailed T-Test t(8) = 4.206 p = 0.003, t(7) = 11.333, p = 0.000, respectively). (**g**) *w* + B group activity in response to 50 Hz and 20 ms of light was significantly different from baseline (One-Sample 2-tailed T-Test t(7) = 3.618, p = 0.009. (**h**) *w* + O group activity in response 30 Hz and 33 ms and 50 Hz and 20 ms of light was significantly different from baseline (One Sample 2-tailed T-Test t(8) = 3.552, p = 0.007, t(7) = 2.746, p = 0.029). *p < 0.05, **p < 0.01, ***p < 0.001.

Interestingly, genetic background had a significant effect on group activity responses to ethanol (Repeated Measures ANOVA with planned contrasts at 140 s, 600 s, and 1100 s, F(6, 56) = 14.499, p = 0.00; Fig. 5a). Wild-type Canton-S (*w* + CS) responses were significantly different than wild-type Berlin (*w* + B) and wild-type Oregon-R (*w* + O) (Posthoc with Bonferroni Corrections p = 0.033–0.000). Flies with a *w-* mutation on the CS background (*w* *−* CS), which are commonly used for behavioral studies, had significantly different activity than all wild-type strains (Posthoc with Bonferroni Corrections p = 0.033–0.000). Most striking was the significant difference in baseline group activity in the absence of alcohol*. w* *−* CS baseline group activity was nearly double that of *w* + CS and four times greater than *w* + B and *w* + O. Previous studies have implicated *w-* in a number of behaviors including courtship^21^ and copulation success^22^, locomotor recovery from anoxia^23^ and phototaxis. These data support an additional role for *w-* in group activity. In line with alcohol sensitivity studies^24^, *w* *−* CS also exhibited a reduction in ethanol induced activity with prolonged exposure.

Initial behavioral studies using *Chrimson*^14^ report that experimental and control flies startle to red light used for optogenetic stimulation. Preliminary data from our lab also suggested that group activity was particularly sensitive to the onset and offset of red light. Thus, we first characterized wildtype group activity responses at different stimulation parameters to identify which parameters elicited the smallest behavioral response (Fig. 5b–d). Using 680 nm of red light, 40 Hz and 25 ms was the only stimulation parameter that did not produce a significant difference in group activity to light onset across all genotypes (One sample t-test: Fig. 5e t(8) = 2.277, p = 0.05, Fig. 5f t(8) = 2.036, p = 0.08, Fig. 5g t(8) = 1.18, p = 0.27, Fig. 5h t(8) = 1.404, p = 0.20). Similarly, 40 Hz and 25 ms did not produce a significant difference in group activity to light offset (Supplementary Fig. 3). Thus, 680 nm of red light pulsed at 40 Hz and 25 ms was selected for all subsequent optogenetic experiments.

### Thermogenetic and optogenetic induced changes in locomotion

Previous inactivation experiments demonstrated that ethanol-induced increases in locomotor activity are dependent on dopamine receptor expression in the R2/R4 neurons that arborize within the central complex’s ellipsoid body (EB)^11^. We sought to replicate this work with increased temporal resolution to identify the specific behavioral role of these neurons in ethanol-induced locomotion. We also identified a causal role for these neurons in locomotor activity by testing whether optogenetically activating these neurons recapitulates the acute locomotor response to ethanol in a group setting. Thus, using the *P-element GAL-4* insertion line *4-67-GAL4* and we validated the flyGrAM platform and explored the effects of thermogenetic inactivation and optogenetic stimulation of specific EB afferents within the central complex on group locomotor activity.

**Figure 6:**
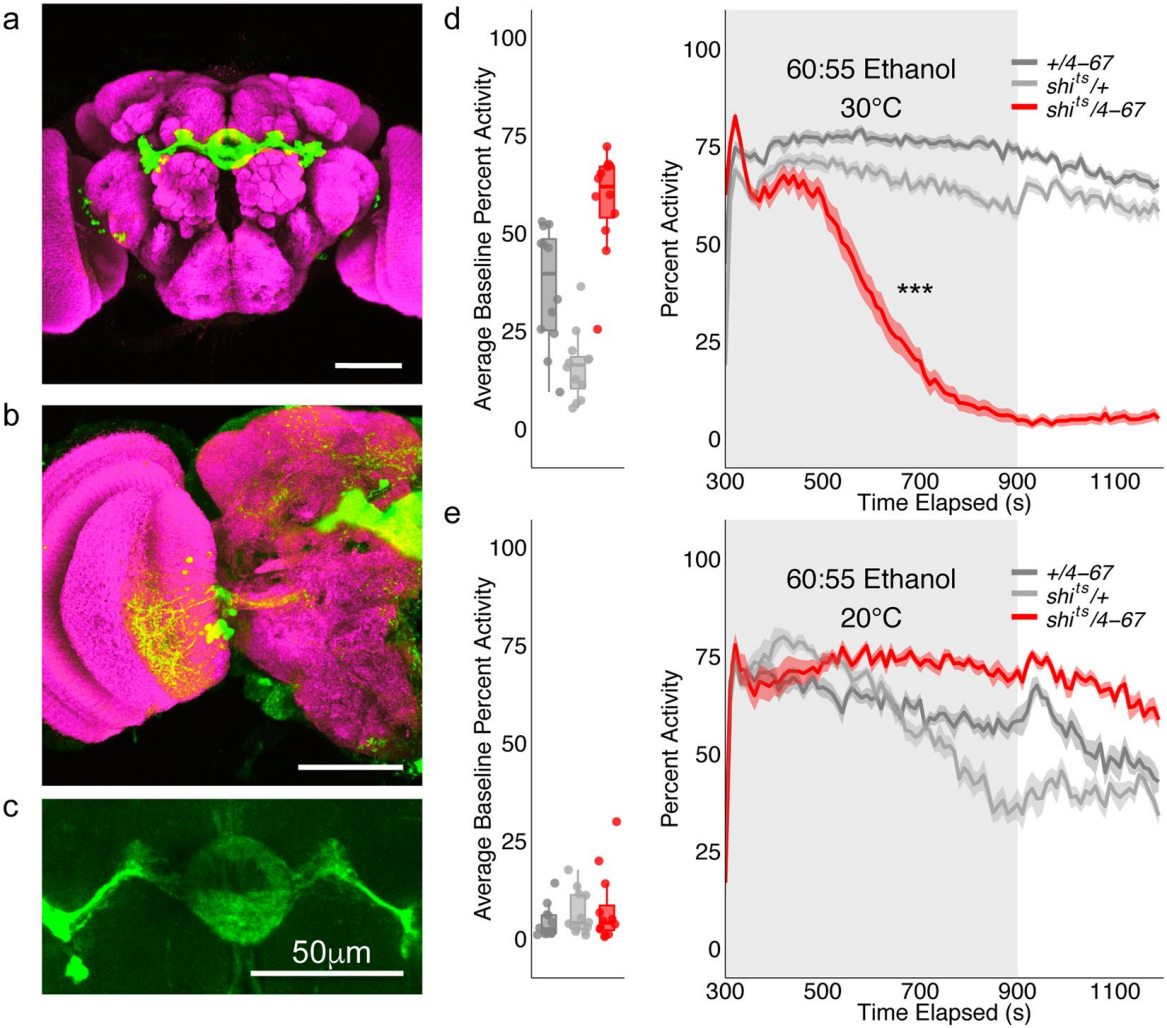
Group locomotor activity in response to ellipsoid body thermogenetic inactivation. (**a**) Anterior view of expression pattern of *4–67-GAL4*. Scale bar is 50 μm. (**b**) Posterior view of expression pattern of *4–67-GAL4*. Scale bar is 50 μm. (**c**) Neuropil expression pattern of *4–67-GAL4* specific to the ellipsoid body. Scale bar is 50 μm. (**d**,**e**) Group activity counts for each genotype were binned over 10 second periods, averaged across biological replicates (+*/4–67* n = 12, +*/shi*^*ts*^ n = 12, *shi*^*ts*^*/4–67* n = 12) of 10 flies each and plotted against time. Ethanol was delivered over a 10-minute period starting at 300 s as denoted by the gray shaded region. Lines with shaded ribbon depict mean +/− standard error. Group activity during baseline was averaged across flies and plotted by genotype. (**d**) Group activity at restricted temperatures was significant reduced in experimental flies (Repeated Measures ANOVA with Greenhouse-Geisser correction F(1.530, 50.485) = 41.896, p = 0.000, Posthoc with Bonferroni correction p = 0.000). (**e**) Experimental flies exhibited normal ethanol-induced locomotor activity at permissive temperatures. ***p < 0.001.

Consistent with previous reports^11^, inactivating R2/R4 EB neurons in the context of intoxicating doses of ethanol significantly reduced locomotor activity (Repeated Measures ANOVA with Greenhouse-Geisser correction F(1.530, 50.485) = 41.896 p = 0.000, Posthoc with Bonferroni correction p = 0.000, Fig. 6a). Strikingly within 5 minutes of ethanol onset, experimental flies began to prematurely sedate. This premature sedation was not a consequence of prolonged exposure to restrictive temperatures (Supplementary Fig. 4). At permissive temperatures, experimental flies exhibited normal alcohol induced locomotor activity (Fig. 6b).

**Figure 7:**
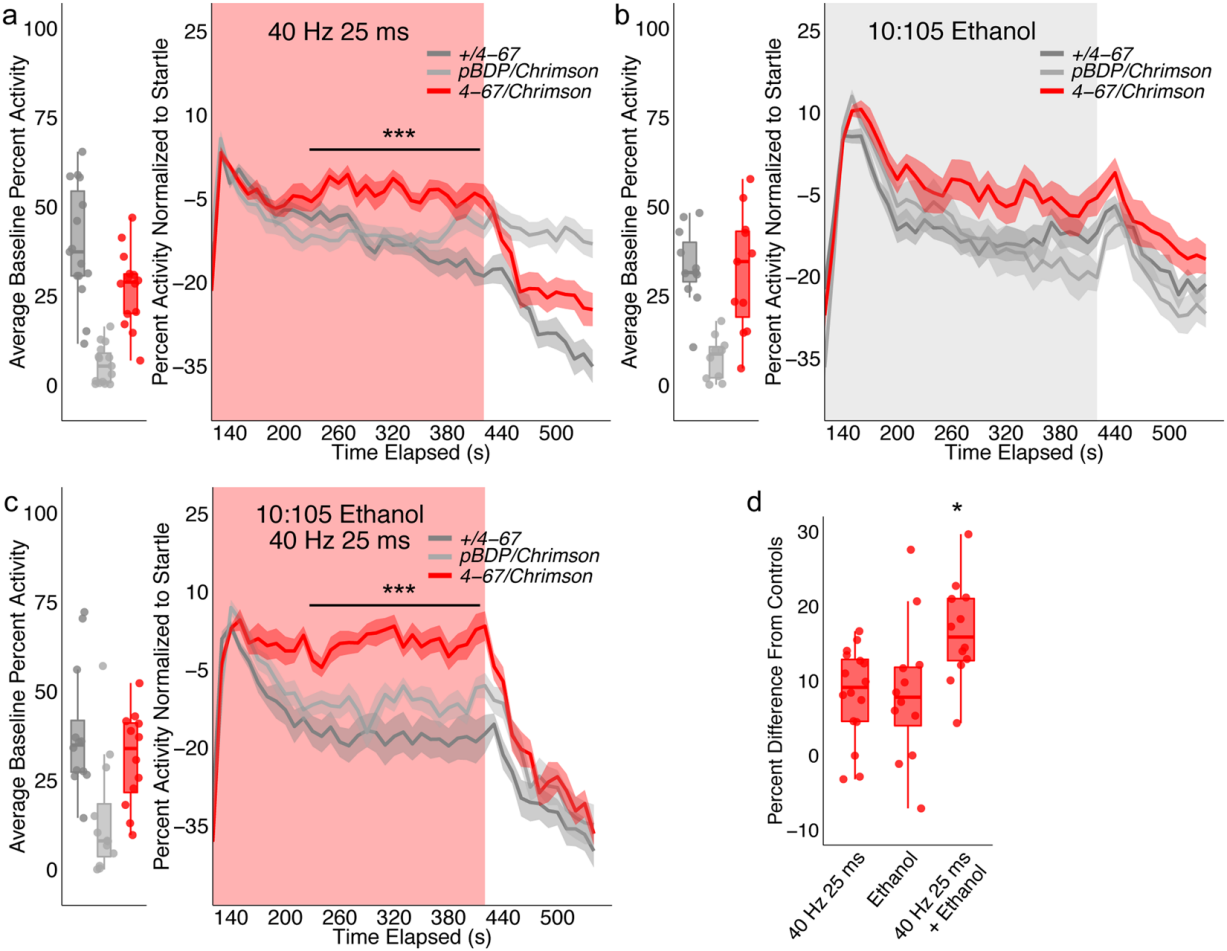
Group locomotor activity in response to ellipsoid body optogenetic stimulation. Group activity counts for each genotype were normalized to 30 seconds following the onset of light and/or ethanol, binned over 10 second periods, averaged across biological replicates of 10 flies each and plotted against time. Red light and/or ethanol was delivered over a 5- minute period starting at 120 s. Lines with shaded ribbon depict mean +/− standard error. Group activity during baseline was averaged across flies and plotted by genotype. (**a**) +*/4–67* n = 16, *pBDP/Chrimson* n = 16, *4–67/Chrimson* n = 16. Group activity during stimulation (200–410 s) was significantly increased in experimental flies (Repeated Measures ANOVA with a Greenhouse-Geisser correction F = (18.894, 425.106) = 3.254, p = 0.000, Posthoc with Bonferroni correction p = 0.001–0.000). (**b**) +*/467* n = 12, *pBDP/Chrimson* n = 12, *4–67/Chrimson* n = 12. Group activity during ethanol exposure (200–410 s) was not significantly different across genotypes (Repeated Measures ANOVA with a Greenhouse-Geisser correction F = (15.607, 249.712) = 1.949, p = 0.018, Posthoc with Bonferroni correction p = 0.063–1.000). (**c**) +*/4–67* n = 12, *pBDP/Chrimson* n = 12, *4–67/Chrimson* n = 12. Group activity during ethanol exposure and optogenetic stimulation (200 s-410 s) was significantly increase in experimental flies (Repeated Measures ANOVA with a Greenhouse-Geisser correction F = (19.432, 310.913) = 1.690, p = 0.035, Posthoc with Bonferroni correction p = 0.008–0.000). (**d**) Group activity as compared to controls significantly increased in response to optogenetic stimulation and ethanol exposure as compared to stimulation alone or ethanol alone (One-way ANOVA F(2, 37) = 5.098, p = 0.011, Posthoc with Bonferroni correction, p = 0.033–0.019). *p < 0.05, ***p < 0.001.

Because data from our lab and others^14^ show that wildtype and control flies startle to red light, group activity for optogenetic experiments was normalized to startle, defined by the first 30 seconds following the onset of light. Interestingly activation alone of R2/R4 neurons produced a sustained, but modest increase in group activity that was significantly different from controls (200 s–410 s; Repeated Measures ANOVA with Greenhouse-Geisser correction F(18.894, 425.106) = 3.254, p = 0.00; Posthoc with Bonferroni correction p = 0.00, Fig. 7a). Considering that the dramatic reduction of locomotor activity in response to R2/R4 neuronal inactivation was specific to the context of ethanol, we reasoned that perhaps robust R2/R4 neuronal activation would also require the context of ethanol. We hypothesized that in the context of low levels of ethanol (10:105 ETOH: Air) control flies would not reach group activity indicative of intoxication, but activation of R2/R4 neurons in the context of these low doses might cause flies to behave as though they were intoxicated. Thus, we selected a non-intoxicating concentration of ethanol to stream into the chamber for five minutes to minimize the effect of ethanol on controls, and used the same activation parameters (40 Hz 25 ms for 600 s) to activate R2/R4 neurons.

Experimental and control flies exhibited similar group activity levels in response to low levels of ethanol (10:105 ETOH: Air, Fig. 7b Repeated Measures ANOVA with Greenhouse-Geisser correction F(15.607, 249.712) = 1.949, p = 0.018, Posthoc with Bonferroni correction p = 0.093 and 0.061). However, strikingly, the activation of R2/R4 neurons in this context produced a robust increase in group activity that was significantly different from controls (Repeated Measures ANOVA with Greenhouse-Geisser correction F(19.432, 310.913) = 1.690, p = 0.010, Posthoc with Bonferroni correction p = 0.008-0.000, Fig. 7c). A comparison of the difference in experimental group activity to controls revealed that the combination of activation and ethanol elicited a significant increase in group activity compared to activation alone or ethanol alone (One-way ANOVA F(2, 37) = 5.098, p = 0.011, Posthoc with Bonferroni correction, p = 0.033-0.019, Fig. 7d), suggesting that the effects of R2/R4 neuronal activation is most robust in the presence of ethanol.

## Discussion

We introduce a new real-time automated assay called the flyGrAM to quantify *Drosophila* group locomotor activity. In order to validate and demonstrate the utility of this behavioral data collection platform, we characterized group locomotor activity responses to two treatments known to change locomotion: ethanol administration and manipulation of ellipsoid body neurons. We successfully demonstrate that the flyGrAM is a sensitive method for quantifying group activity changes in response to environmental manipulations and show how group activity response profiles differ in response to different ethanol concentrations. Further, we show that optogenetic activation of R2/R4 ring ellipsoid body (EB) neurons similarly increase locomotor group activity consistent with their identified role in modulating ethanol induced hyperactivity. A low error rate and close match with manually annotated group activity values demonstrate that the flyGrAM is a robust means of automatically quantitating group activity in a variety of paradigms. Thus, the flyGrAM is a straightforward, cost-effective, and versatile activity tracking system.

### Assessing locomotor activity changes

Ethanol’s effect on locomotor activity is well characterized in *Drosophila*. Previous studies quantified ethanol’s locomotor effects using a beam crossing method^25^ and a video analysis method (Dynamic Image Analysis System or DIAS)^5,20,26–28^. These established approaches for looking at alcohol locomotor responses provide useful benchmarks to assess flyGrAM performance. With respect to beam crossing methods, the flyGrAM platform has the distinct advantage of being able to detect all activity in given behavioral apparatus while also allowing reliable quantification of group activity. With respect to other video tracking systems, flyGrAM has the advantage of producing data in real time, which permits quick data collection and analysis and may be adjusted for use in a closed-loop system where behavior of the animals can control instrumentation.

A comparison of the results from the flyGrAM and the inebri-actometer^25^ show similar activity profiles in response to ethanol. Ethanol induced activity is characterized by a sharp increase in activity followed by sedation. Because of the improved spatial coverage, the flyGrAM is able to uncover more pronounced startle responses to ethanol compared to the inebri-actometer. Importantly, sustained group activity increases are not merely a consequence of odor presentation. When flies are introduced to an odor instead of intoxicating doses of ethanol, group activity sharply increases at odor onset, but quickly returns to baseline for the duration of odor presentation. The DIAS system^26–28^, in contrast, quantifies movement parameters such as fly speed, heading, etc. It cannot, however, perform real-time analysis. It is also not open source nor actively maintained, which makes software acquisition challenging. Despite not collecting specific movement parameters, analysis with both the flyGrAM and DIAS show similar trends. Low ethanol concentration data shows a biphasic startle period, followed by a slow ramping of activity in response to ethanol. The cessation of ethanol also resulted in a slight increase in group activity under low ethanol. This coincident increase is likely due to the momentary change in air pressure from switching off ethanol and is a subtle effect not seen in previous DIAS results, which used an identical ethanol delivery system^5^. High ethanol concentrations revealed a triphasic component corresponding to ethanol sedation. Flies exposed to high concentrations of ethanol (100:15 and 110:05) failed to maintain increased locomotor activity in response to cessation of ethanol delivery, suggesting sedation. These results demonstrate that the flyGrAM is a sensitive and robust means of quantifying group activity in response to ethanol.

### Assessing circuitry for ethanol-induced activity changes

The ellipsoid body is central brain structure within the central complex that has an established role in visual processing, including visual orientation^29–31^ and spatial memory^32–37^. Recent work has also identified the importance of ellipsoid body neurons in regulating ethanol sensitivity^38^, tolerance^39^, as well as ethanol-induced hyperactivity^11^. Specifically expressing *Slo*, a BK type Ca^2+^ activated K^+^ channel, in the EB increased resistance to sedation by opposing alcohol-induced decreases in neural excitability^38^. Similarly, *DopR* mutants were reported to display blunted ethanol-induced hyperactivity that was only restored with selective expression of D1-like dopamine receptor DopR in R2 and/or R4 ring neurons innervating the EB of the central complex^11^. Perhaps dopamine also works to increase excitability of R2/R4 neurons in the context of alcohol. Interestingly, dopamine also seems to modulate motor activity and turning behaviors in the absence of alcohol highlighting the importance of dopamine in goal directed locomotion.

R2/R4 ring neurons are a subclass of large-field neurons that have postsynaptic connections in the lateral triangle and arborize presynaptically in concentric rings of the EB^40^. Recently, Wolff, *et al*.^41^ comprehensively described the complex, yet highly organized connections between the protocerebral bridge and the EB within the central complex, whereby protocerebral bridge projections largely target separate EB wedges in a near 1:1 correspondence. Further there is considerable evidence of strong tuning in subsets of ring neurons innervating the EB to localized visual features as well as a defined role for the EB in stimulus selection^42–44^. Functional connections between the protocerebral bridge and EB are thought to support a head direction system and thus egocentric navigation^42,43,45–47^. It’s possible that the blunting ethanol-induced hyperactivity previously reported^11^ and premature sedation described here is a consequence of severe disruptions to the dynamics of the ring attractor network^45,46^ and thus orientation and goal-directed locomotion.

**Figure 8:**
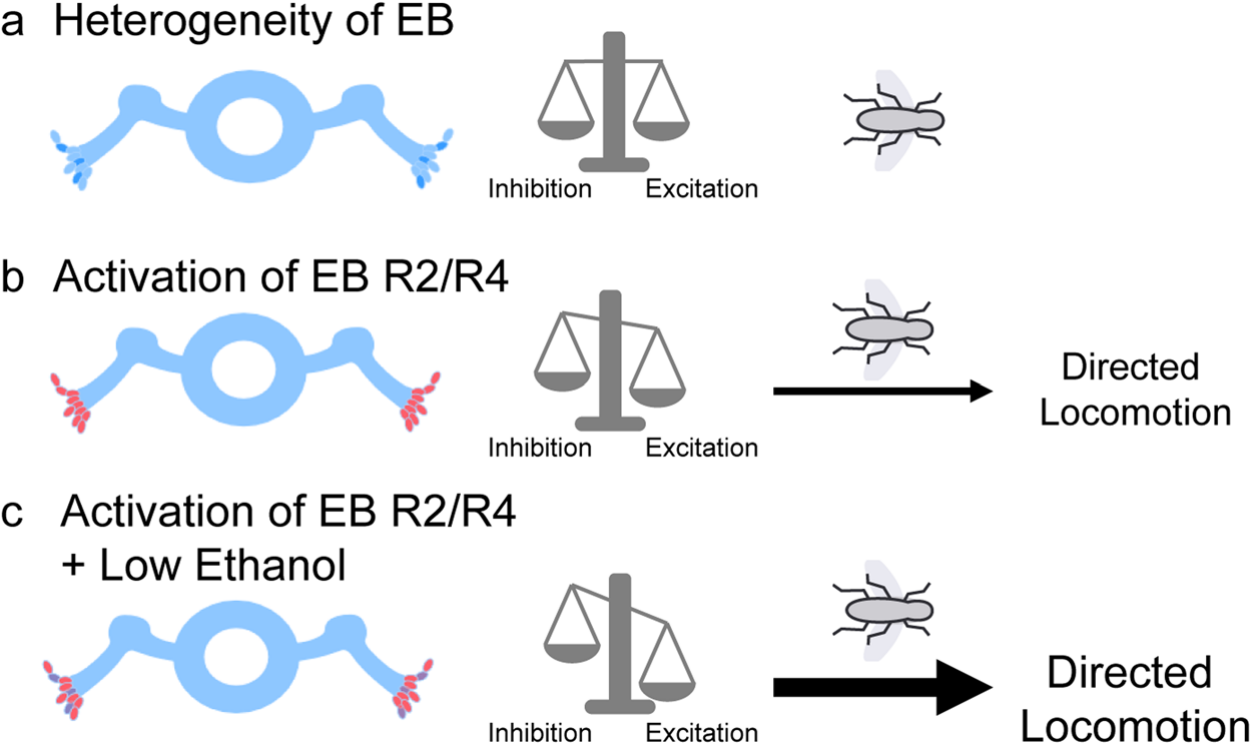
Model of ellipsoid body (EB) role in ethanol-induced locomotor activity. (**a**) Heterogeneity exists in EB that balances excitation and inhibition of locomotor activity. (**b**) Activation of EB R2/R4 neurons results in the activation of a population of EB which shifts balance towards excitation thereby promoting locomotor activity. (**c**) Activation of EB R2/R4 neurons and low levels of ethanol results in the same activation of EB neurons, however the presence of low levels of ethanol decrease neural excitability of subpopulations of EB neurons likely reducing inhibition allowing for an even greater shift in excitation thereby promoting enhanced locomotor activity.

Previous work demonstrated that the EB comprises primary GABAergic neurons^48,49^, however, recent work has suggested that perhaps the EB is more heterogenous than previously thought, both in their molecular identity and their postsynaptic targets^50,51^. We show that expressing a well-characterized red-shifted channelrhodopsin called *Chrimson*^14^ in R2/R4 neurons of the EB increase group locomotor activity in response to 40 Hz and 25 ms pulses of 680 nm wavelength. Interestingly, increases in group locomotor activity was most robust in the context of low levels of ethanol. We propose that heterogeneity within the ellipsoid body that allows for the balance of inhibition and excitation might underlie these differences (Fig. 8). When R2/R4 neurons are activated, the balance between inhibition and excitation is shifted and the flies increase their group locomotor activity. This is consistent with previous reports that activation of subsets of R2/R4 neurons increases walking behaviors^52^. However, in the context of alcohol it’s possible that subpopulations of EB neurons are more sensitive to alcohol-induced decreases in neural excitability. Given that neurons express GABA_A_ receptors are highly expressed in the mushroom body and EB^53^, it’s possible these subpopulations are GABAergic. This sensitivity likely reduces inhibition and allows the artificial activation of R2/R4 neurons to have a more robust behavioral consequence. Further work is needed to better understand the impact of ethanol on the populations of EB neurons and how this disruption relates to locomotor activity.

### Extending the flyGrAM platform

Although we have successfully demonstrated two possible uses for the flyGrAM in this report, there exist a number of *Drosophila* locomotor activity paradigms that would benefit from a real-time, cost-effective, and accessible group activity quantification platform. One obvious extension of the flyGrAM would be to use it for quantifying locomotion in response to other drugs of abuse and for behavioral phenomena such as drug sensitization. The ease with which optogenetic stimulation or thermogenetic activation can be added to an experiment also opens up interesting possibilities for assessing the necessity and sufficiency of neural pathways mediating drug locomotor effects.

Another area of *Drosophila* research that might benefit from the flyGrAM platform is the study of *Drosophila* circadian rhythms in social settings. Although methods for determining the activity profile of single flies are well characterized, there exist few methods for assessing circadian rhythms for groups of flies. Previous work examining socio-sexual consequences on circadian rhythm has resorted to group housing flies in mixed or same-gender settings before assaying individuals for differences in circadian rhythms^54^. As previously mentioned, using the flyGrAM we have successfully recorded group activity of flies for at least 24 hours, thus the flyGrAM provides the powerful opportunity to directly quantify the activity of heterogenous populations across time.

Future work with the flyGrAM platform could focus on extending its capabilities for studying more advanced behaviors. For example, with the appropriate hardware and software modifications, it would be possible to implement locomotor or location dependent delivery of optogenetic or drug stimuli. This would open up new possibilities for using the flyGrAM to characterize *Drosophila* behavior in classical conditioning or operant self-administration paradigms.

### Use of flyGrAM in an educational setting

The ease of setting up and using the flyGrAM makes it particularly useful in an educational setting such as a laboratory classroom. Using this platform, students have the opportunity to learn the basic principles of: 1) *Drosophila* genetics, 2) optogenetics, 3) constructing a tracking apparatus, 4) using computer vision algorithms to track animals, and 5) rigorous quantification of behavior data. Our experience using this platform in the Cold Spring Harbor Laboratory (CSHL) Neurobiology of *Drosophila* course suggests that it is an effective tool for exploring the neural basis of behavior in a laboratory classroom setting.

## Conclusions

In this paper, we have introduced a new platform for quantifying *Drosophila* group activity called the flyGrAM. This platform uses an inexpensive and simple to use video tracking methodology to quantify group locomotor activity in real-time. Unlike computationally expensive, video-based group analyses, flyGrAM provide an instant readout of group activity by not maintaining the identity of individual flies and calculating measurements such as distance traveled or speed. We have validated this platform by using it to assess group activity in response to known locomotion modulating stimuli such as ethanol as well as thermogenetic manipulation, and optogenetic stimulation of EB neurons. We also provide examples of offline advanced post processing of the flyGrAM raw video output using C-trax^9^ to track individual flies and calculate behavioral features. Our results demonstrate that the flyGrAM platform is a robust means of obtaining an online read out of group activity, while maintaining the potential for further behavioral analyses using advanced post-processing platforms. We anticipate that the versatility of the flyGrAM platform will make it central to studying a number of different locomotor behaviors.

## Supplementary information

Supplementary File

Supplementary Video 1

## Data Availability

All datasets generated in the flyGrAM and reported here are available for download in the flyGrAM repositories (https://github.com/kaunlab).
