# Supplementary material for: Automated real-time quantification of group locomotor activity in Drosophila melanogaster
Source: Sci Rep. 2019 Mar 14;9:4427. doi: 10.1038/s41598-019-40952-5 (PMC6418093; doi:10.1038/s41598-019-40952-5)
Supplement: Supplementary file 1 — Supplementary File [file 41598_2019_40952_MOESM1_ESM.pdf]

Supplementary Information

Automated real-time quantification of group locomotor activity in *Drosophila melanogaster.*

Kristin M. Scaplen[1], Nicholas J. Mei[1], Hayley A. Bounds[1], Sophia L. Song[1], Reza Azanchi[1], Karla R. Kaun[1]

[1]Department of Neuroscience, Brown University, Providence, Rhode Island, 02912, USA.

Table of Contents:

Supplementary Figure 1: Schematic for laser cutting FlyGrAM chambers……………………………….. 2
Supplementary Figure 2: 24 hour group activity plots……………………………………………………….. 3
Supplementary Figure 3: Percent difference in group activity from light offset from baseline…. 4
Supplementary Figure 4: Group locomotor activity in response to ellipsoid body inactivation…. 5
Supplementary Table 1: Detailed list of component parts for FlyGrAM construction………………. 6
Supplementary Video 1 legend: Real-time activity detection during air and ethanol exposure... 7

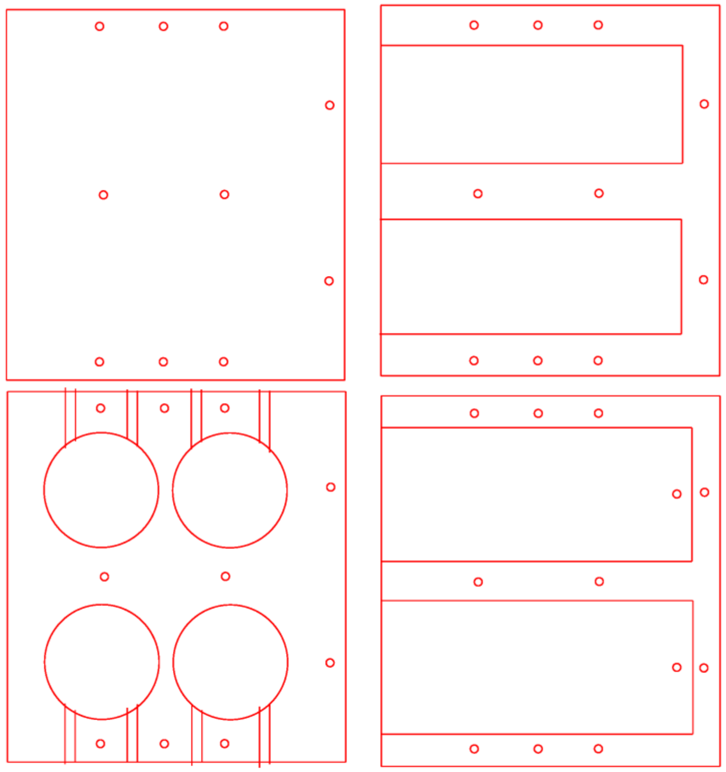

Supplementary Figure 1. Schematic for laser cutting FlyGrAM chambers.

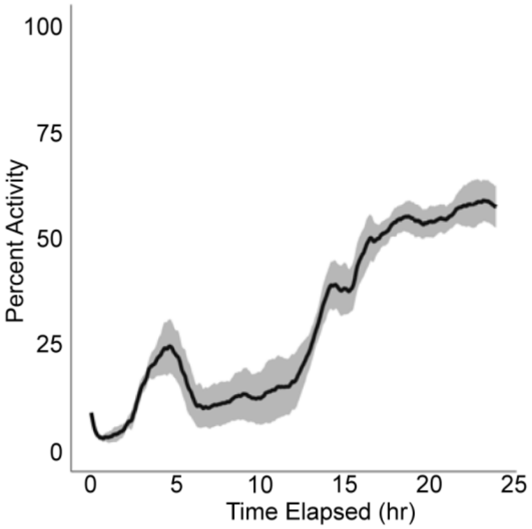

Supplementary Figure 2. Group locomotor activity plots of flies recorded for 24 hours. n=4 (10 flies each).

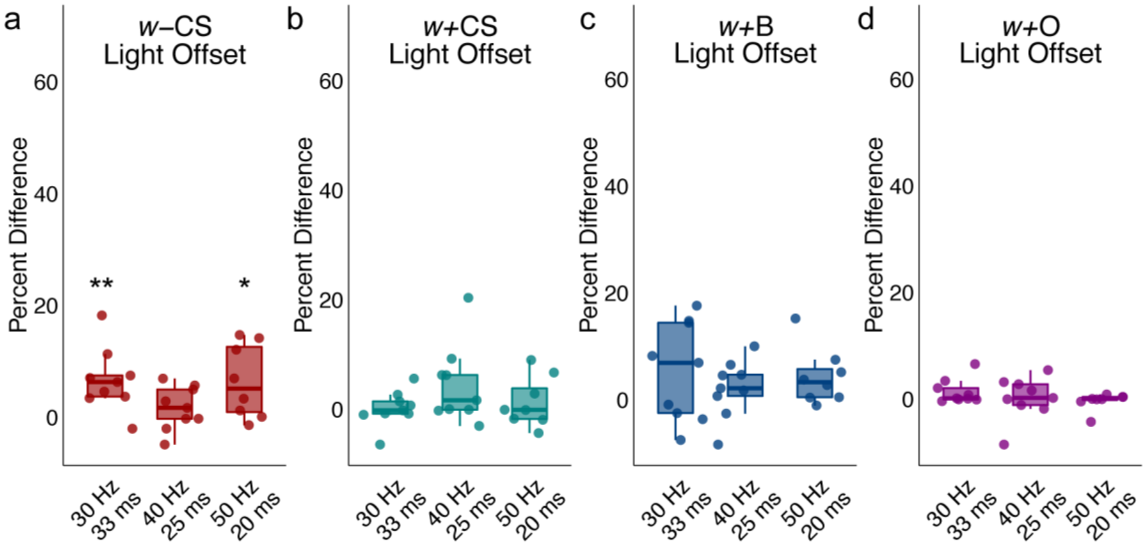

Supplementary Figure 3. Percent difference in group activity from light offset (420-450 s) as compared to baseline (500-530 s) of red light (680 nm) at 30 Hz and 33 ms, 40 Hz and 25 ms, and 50 Hz and 20 ms. (a) *w*-CS group activity in response to 30 Hz and 33 ms and 50 Hz and 20 ms of light was significantly different from baseline (One-Sample 2-tailed T-Test $t(8) = 3.554$, $p = 0.007$, $t(7) = 2.777$, $p = 0.27$, respectively). 40 Hz and 25 ms was not significantly different from baseline (One-Sample 2-tailed T-Test $t(8) = 1.286$, $p = 0.234$) (b) *w+*CS group activity in response to 30 Hz 33 ms, 40 Hz and 25 ms, and 50 Hz and 20 ms of light offset was not significantly different from baseline (One-Sample 2-tailed T-Test $t(8) = 0.141$ $p = 0.891$, $t(8) = 1.893$, $p = 0.095$, $t(7) = 0.814$, $p = 0.442$). (c) *w+*B group activity in response to 30 Hz and 33 ms, 40 Hz and 25 ms, and 50 Hz and 20 ms of light offset was not significantly different from baseline (One-Sample 2-tailed T-Test $t(8) = 1.722$, $p = 0.123$, $t(8) = 1.226$, $p = 0.255$, $t(7) = 2.332$, $p=0.052$). (d) *w+*O group activity in response to 30 Hz and 33 ms, 40 Hz and 25 ms, and 50 Hz and 20 ms of light offset was not significantly different from baseline (One-Sample 2-tailed T-Test $t(8) = 1.835$ $p = 0.104$, $t(8) = 0.159$ $p = 0.877$, $t(7) = -0.641$ $p = 0.542$).

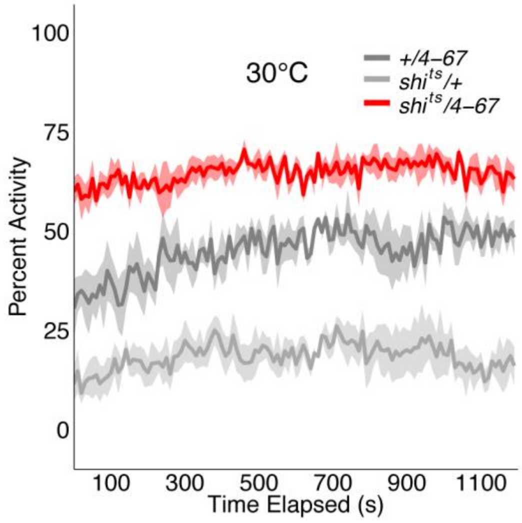

Supplementary Figure 4. Group locomotor activity in response to ellipsoid body thermogenetic inactivation in the absence of any stimuli.

Component	Part Number	Vendor	Approx. Cost (in USD 2018)
Acrylic Plates for Arenas / IR Light Diffuser	0.118" thick plates in various colors	Various	15
Adaptor Lens	PT-0620	M12 Lenses	5
Arduino Uno	UNO	Arduino Foundation	25
BuckPuck (for optogenetics)	3023-D-E-1000P	LuxDrive	15
Camera IR Filter	#54-664 (RG-850)	Edmund Optics	25
Camera Mounting Stand	20mm x 20mm T-Slotted Extrusion x 305mm	80/20 Inc.	5
IR Array Circuit Board	27520 **[1]**	Sodial	5
IR Array Circuit Board Standoffs	a11070700ux0190	uxcell	15
IR LEDs	SFH 487-2 **[2]**	Osram	70
LED Array Heat Sink	TO-220 Heat Sink	Radio Shack	5
Nylon Hex Nut (Arena Fastener)	94812A200	McMaster-Carr	5
Nylon Socket Head Screws (Arena Fastener)	95868A261	McMaster-Carr	5
Red LED Array (for Optogenetics)	1125-1089-ND	Digi-Key	10
USB Camera	USBFHD01M-L21	ELP	45
Wires (Black and Red)	24UL1007STRBLA (Black) and 24UL1007STRRED (Red)	Remington	20
2N3906 transistor	N/A	Various	5
5.1K Ohm resistors	N/A	Various	5
12V AC --> DC Converter	N/A	Various	5
9V AC --> DC Converter	N/A	Various	5

Supplementary Table 1. Detailed list of component parts for FlyGrAM construction.

Supplementary Video 1. Example video of real-time activity detection during air and ethanol exposure. The first 48 seconds shows tracking during air exposure whereas the last 20 seconds shows tracking during ethanol exposure. In rare cases, two moving flies that are too close can be merged into a single detection, or single flies which make sudden decelerations can be separated into two detections.
